# Retinal Microvascular Change in Hypertension as measured by Optical Coherence Tomography Angiography

**DOI:** 10.1038/s41598-018-36474-1

**Published:** 2019-01-17

**Authors:** Woo Hyuk Lee, Jae-Hyeong Park, Yeokyoung Won, Min-Woo Lee, Yong-Il Shin, Young-Joon Jo, Jung-Yeul Kim

**Affiliations:** 1 Department of Ophthalmology, Chungnam National University College of Medicine, Daejeon, Republic of Korea; 2 Department of Cardiology in Internal Medicine, Chungnam National University College of Medicine, Daejeon, Republic of Korea

## Abstract

Many studies have reported the effect of hypertension on microcirculation of the retina. Advance of optical coherence tomography angiography (OCTA) allows us more detailed observations of microcirculation of the retina. Therefore, we compared OCTA parameters between chronic hypertension (disease duration of at least 10 yrs; Group A, 45 eyes), relieved hypertensive retinopathy (grade IV HTNR < 1 yr prior; Group B, 40 eyes), and normal controls [Group C (50 eyes) ≥ 50 yrs old and Group D (50 eyes) < 50 yrs old]. A 3 × 3 mm macular scan was performed in each group by OCTA. In vessel density of 3 mm full, group A and B were significantly decreased compared to normal control group (Group A *vs*. C; 19.4 mm^−1^ *vs*. 20.1 mm^−1^, Group B *vs*. D; 19.8 mm^−1^ *vs*. 21.8 mm^−1^, all p < 0.05). In foveal avascular zone, group A and B were significantly increased compared to normal control group (Group A *vs*. C; 0.35 mm^2^ *vs*. 0.30 mm^2^, Group B *vs*. D; 0.36 mm^2^ *vs*. 0.29 mm^2^, all p < 0.05). OCTA is useful for examining retinal microcirculatory changes in hypertension and we confirmed that hypertension affects the OCTA parameters. Considering the effect of hypertension on the change of microvasculature, care is required in the interpretation of OCTA parameters in various ophthalmic condition.

## Introduction

The World Health Organization has reported that hypertension affects 1 billion patients worldwide^1^. Screening for hypertension is very important as it is a major risk factor for cardiovascular disease and mortality^2^. Hypertension elevates the systemic arterial pressure and peripheral vascular resistance and causes microvascular changes that can be examined directly in the eye. Signs of retinal damage caused by hypertension can be observed before target organ damage begins to manifest clinical symptoms in hypertensive patients. Thus, the vascular changes in the retina may be useful indicators of target organ damage in hypertensive patients^3,4^. Fundus changes caused by hypertension include arteriolar narrowing, arteriovenous nicking, cotton wool spots, intraretinal hemorrhage, and papilledema. These changes can be observed using various methods, including fundus examination, optical coherence tomography (OCT), and fluorescence angiography.

There have been a number of studies of hypertension-induced retinal microvascular changes. Wong *et al*.^5^ used digital retinal photography and imaging software to measure retinal vessel widths to objectively quantify generalized arteriolar narrowing. Von *et al*.^6^ reported that elevation of mean arterial blood pressure was strongly correlated with reduced central retinal artery caliber and increased central retinal vein caliber. Another study indicated a strong correlation between hypertension and retinal microvascular caliber^7^.

Advances in spectral-domain (SD) OCT have allowed more detailed observations of retinal structure. An SD-OCT-based study indicated that retinal vessel diameter measurements obtained with SD-OCT are highly reproducible and vessel walls are significantly thickened in systemic hypertension^8^. In addition, our previous study showed that severe hypertensive retinopathy (HTNR) and chronic hypertension (HTN) cause reductions in thickness of the retinal nerve fiber layer and central macula over time^9,10^. However, the effect of hypertension on microcirculation of the retina is unclear.

Optical coherence tomography angiography (OCTA) is a recently developed noninvasive diagnostic imaging technique that employs motion contrast extracted from high-speed OCT images to produce depth-resolved, high-resolution images of retinal and choroidal vasculature without dye injection^11^. We postulated that this new diagnostic tool could be used for retinal vascular analysis in patients with hypertension. Recently, commonly available OCTA instruments allow quantification of vessel density (VD), perfusion density (PD), and foveal avascular zone (FAZ) area. To our knowledge, there have been no previous studies of hypertensive patients using OCTA. This study was performed to assess the effects of hypertension on changes in the retina vessels and flow in patients with long-term hypertension and relieved HTNR, by comparing OCTA parameters with normal control eyes.

## Materials and Methods

This cross-sectional study was approved by the Institutional Review Board of Chungnam National University Hospital. All procedures were performed in accordance with the tenets of the Declaration of Helsinki. The Institutional Review Board also approved the collection of data from the medical charts of patients with and without retinal diseases. Prior written informed consent was obtained from all subjects after a detailed explanation of the nature of the study and possible consequences associated with participation therein.

### Materials

Patients who visited our retinal clinic between June 2017 and December 2017 were included in the study. Patients without history of intraocular surgery (except non-complicated cataract surgery), ocular trauma, diagnosis of other ophthalmic diseases (except diagnosis with grade IV HTNR, according to the Keith–Wagener–Barker classification system^12^) and systemic diseases in addition to hypertension (e.g., diabetes, dyslipidemia, and stroke) were included.

The patients were divided into the following two groups: a chronic HTN group consisting of patients with hypertension for at least 10 years but without HTNR (group A, 45 eyes), and a relieved HTNR group consisting of patients diagnosed with grade IV HTNR TN1 year previously but who have relieved HTNR at the time of the study (group B, 40 eyes).

Patients in group A and B had well-controlled blood pressure did not exhibit acute HTNR changes at the time of the study. (e.g., cotton wool spots, intraretinal hemorrhage, exudative retinal detachment, or papilledema). The normal control group consisted of subjects with no medical history of systemic or ophthalmological disease. To minimize the effect of age on OCTA parameters, we divided the normal control group into two subgroups according to age, as groups A and B showed a significant difference in age (group A: 60.6 ± 6.7 years; group B: 41.9 ± 8.8 years, *p* < 0.001). Control subjects ≥ 50 years old were classified into group C (50 eyes), and those < 50 years old were classified into group D (50 eyes).

In all groups, one eye was selected randomly. Including SD-OCT and OCTA tests, we performed standard eye examinations, and measured best corrected visual acuity (BCVA), Spherical equivalent, axial length, and intraocular pressure (IOP) using noncontact tonometry, fundoscopy, and fundus photography. The eyes with axial length < 23.60 mm or m 25.55 mm, high degree of astigmatism (more severe than ± 3 diopters), and BCVA < , 20/25 were excluded.

### OCT and OCTA measurement

Before the examinations, all patients used a mydriatic agent (tropicamide 5 mg/mL and phenylephrine HCL 5 mg/mL, Mydrin-P; Santen Pharmaceutical, Osaka, Japan) three times per 5 minutes and anesthetic eye drops (proparacaine hydrochloride 0.5%, Alcaine; Alcon, Fort Worth, TX) to minimize blinking. After full mydriasis, an experienced examiner performed the examination twice (5-minute interval) in the same position using a Cirrus HD-OCT 5000 instrument with AngioPlex software (Carl Zeiss Meditec, Dublin, CA) (with AngioPlex software; ver. 10.0; Carl Zeiss Meditec, Jena, Germany) to acquire microvasculature images of the macular area (3 × 3 mm).

This instrument provides high-resolution microvascular images of the retina and choroid via 68,000 A-scans per second at a center wavelength of 840 nm. To minimize motion artifacts, FastTrack retinal tracking technology was used. When the 3 × 3 mm scan pattern was used, there were 245 A-scans in each B-scan along the horizontal dimension and 245 B-scan positions along the vertical dimension. Therefore, each A-scan and B-scan is separated by 12.2 μm. Each B-scan is repeated four times at the same position. The instrument provides sensitivity and accuracy by incorporating the optical microangiography (OMAG) algorithm and retinal tracking technology. All scans were analyzed using en face OCTA images generated automatically by the OMAG algorithm in the Cirrus OCTA software. The vascular images of the superficial capillary plexus (SCP) spanned from the internal limiting membrane to the inner plexiform layer. This system allowed quantification of VD, PD, and FAZ only in the SCP.

**Figure 1:**
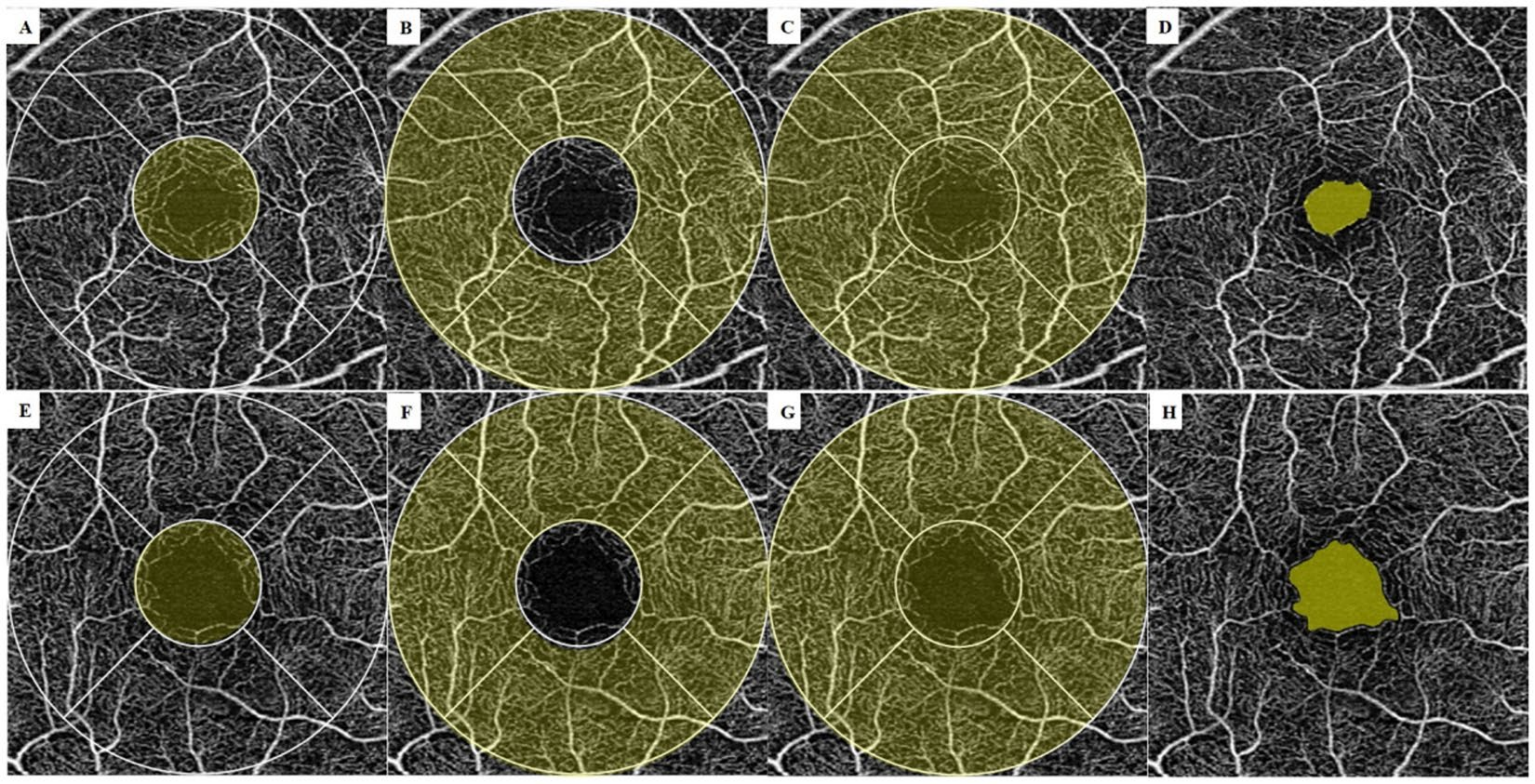
Representative optical coherence tomography angiography (OCTA) images with the signal strength 10. Upper row (**A–D**) are images of normal 60-year old female. Lower row (**E–G**) are images of 60-year old female with chronic hypertension (HTN). Vessel and perfusion density 1 mm center of the two participants were 13.6 mm^−1^, 0.230 (**A**) and 7.8 mm^−1^, 0.126 (**E**); vessel and perfusion density inner ring were 22.1 mm^−1^, 0.393 (**B**) and 21.6 mm^−1^, 0.385 (**F**); vessel and perfusion density 3 mm full were 21.2 mm^−1^, 0.375 (**C**) and 20.5 mm^−1^, 0.361 (**G**), respectively. The automatically detected foveal avascular zone areas were 0.16 mm^2^ (**D**) and 0.39 mm^2^ (**H**), respectively.

The measurement area of the 3 × 3 mm scan was divided into five subfields composed of a 1 mm center and four quadrant sectors (superior, inferior, nasal, and temporal) that were identical to the inner circles of the Early Treatment Diabetic Retinopathy Study subfields. VD (defined as the total length of perfused vasculature per unit area in a region of measurement) and PD (defined as the total area of perfused vasculature per unit area in a region of measurement) of each subfield were measured automatically. FAZ area (Fig. 1D,H) was also measured automatically. VD and PD calculated from a 1 mm diameter circle centered on macula were defined as VD and PD 1 mm center (Fig. 1A,E). VD and PD calculated from 4 region of parafoveal ring were defined as VD and PD inner ring (Fig. 1B,F), VD and PD calculated from a 3 mm diameter circle centered on macula were defined as VD and PD 3 mm full (Fig. 1C,G). All scans were reviewed individually by two investigators (YIS and JYK) for quality assessment (i.e., signal strength, loss of fixation, segmentation error, and motion artifacts), and substandard images were excluded. To obtain clear images through clean medium, those with signal strength ≤ 8 were excluded. Among the OCTA parameters, VD 1 mm center, VD inner ring, VD 3 mm full, PD 1 mm center, PD inner ring, PD 3 mm full, and FAZ area were used for comparisons.

The central foveal thickness (CFT), mean ganglion cell inner plexiform layer thickness (GC-IPL), and average retinal nerve fiber layer thickness (RNFL) were also measured using a 512 × 128 macular cube combination scan mode and a 200 × 200 optic disc cube scan mode.

### Statistical analysis

Various clinical factors including age, sex, laterality, BCVA, IOP, axial length, and refractive error, were compared between groups A and C and between groups B and D using the χ^2^ test and Student’s t test. OCT parameters, the VD, PD, FAZ area, CFT, GC-IPL, and RNFL were also compared between groups A and C and between groups B and D. The average value of two measurements was used in the comparison. Student’s *t* test was utilized for comparison of each measurement. Linear regression analysis was used to investigate the correlations between OCT and OCTA parameters.

All statistical analyses were performed using SPSS for Windows statistical software (ver. 18.0; SPSS Inc., Chicago, IL). Snellen BCVA results were converted to the logarithm of the minimum angle of resolution value (logMAR). Continuous variables are presented as means ± standard deviation. In all analyses, *p* < 0.05 was taken to indicate statistical significance.

The intraclass correlation coefficients (ICC) were calculated to determine the repeatability of consecutively measured OCTA. ICC is the correlation between two variables measured at different time points (t), with values ranging from 0 to 1. As the ICC value approaches 1, the repeatability of the measurement increases proportionally^13^.

## Results

### Demographics

Initially, 201 eyes of 201 patients were included in this study, but 16 eyes were excluded (Group A: 8 eyes; Group B: 6 eyes; Group D: 2 eyes). Even if the signal strength of the OCTA measurement exceeded 8, data affected by motion artifacts or auto-segmentation errors were excluded from the study by agreement between the authors (YIS and JYK).

Finally, 185 eyes of 185 patients (96 males, 89 females) were enrolled in the study. Groups A, B, C, and D consisted of 45, 40, 50 and 50 eyes, respectively. In SD-OCT examination, none of the subjects exhibited any characteristics of HTNR (e.g., subretinal fluid, intraretinal fluid, optic disc edema, or hyper-reflective foci). There was no significant difference in age between groups A and C (*p* = 0.877) or groups B and D (*p* = 0.327). There were also no significant differences in sex, laterality, IOP, axial length, BCVA, or mean spherical equivalent between groups A and C or between groups B and D (Table 1).

**Table 1:** Demographics and clinical characteristics of the subjects.

	Group A	Group C	*p*-value	Group B	Group D	*p*-value
	*n* = 45	*n* = 50		*n* = 40	*n* = 50	
Age (mean ± SD, years)	60.6 ± 6.7	60.8 ± 8.1	0.877^*^	41.9 ± 8.8	40.0 ± 6.0	0.327^*^
Sex (male/female)	25/20	24/26	0.399^†^	20/20	27/23	0.714^†^
Laterality (OD/OS)	22/23	30/20	0.398^†^	20/20	21/29	0.461^†^
BCVA (mean ± SD, LogMAR)	0.00 ± 0.01	0.00 ± 0.02	0.562^*^	0.01 ± 0.02	0.00 + 0.02	0.330^*^
SE (mean ± SD, diopters)	0.2 ± 0.8	0.2 ± 1.0	0.731^*^	−0.1 ± 0.9	−0.2 ± 1.0	0.705^*^
IOP (mean ± SD, mmHg)	15.5 ± 2.6	14.9 ± 2.8	0.332^*^	15.6 ± 2.4	14.7 ± 2.6	0.211^*^
Axial length (mean ± SD, mm)	23.9 ± 0.3	23.8 ± 0.3	0.497^*^	24.0 ± 0.3	24.0 ± 0.4	0.938^*^

-BCVA = best corrected visual acuity; SE = spherical equivalent; IOP = intraocular pressure

-Group A = chronic hypertension group, Group B = relieved hypertension group, Group C = older healthy group, Group D = younger healthy group

-^*^Student’s *t* test

-^†^Chi-squared test.

### OCTA parameters & OCT parameters

In VD and PD analyses, VD 3 mm full of group A (19.4 ± 1.4 mm^−1^) and B (19.8 ± 1.6 mm^−1^) were significantly reduced compared to normal control groups [Group C (20.1 ± 1.0 mm^−1^; *p* = 0.005) and D (21.8 ± 0.8 mm^−1^; *p* < 0.001)]. And, 3 mm full of group A (0.35 ± 0.02) and B (0.37 ± 0.03) in PD were also significantly lower than that of normal control groups [Group C (0.37 ± 0.01; *p* = 0.002) and D (0.39 ± 0.01; *p* = 0.015)]. These trends were also observed in the 1 mm center and inner ring (all *p* < 0.05).

In FAZ area analyses, FAZ area of group A (0.35 ± 0.05mm^2^) and B (0.36 ± 0.03mm^2^) were significantly increased compared to that of normal control groups [Group C (0.30 ± 0.07mm^2^, *p* < 0.001) and D (0.29 ± 0.06mm^2^, *p* = 0.001)].

OCT parameters (CFT, GC-IPL and RNFL) in group A were significantly decreased than groups C (CMT; 264.1 *vs*. 272.0, *p* < 0.020, GC-IPL; 79.1 *vs*. 85.0, *p* < 0.024, RNFL; 93.1 *vs*. 96.7, *p* = 0.025, respectively). This trend was also observed between group B and D (CMT; 253.4 *vs*. 277.3, *p* = 0.001, GC-IPL; 72.7 *vs*. 85.75, *p* < 0.001, RNFL; 91.3 *vs*. 98.2, *p* = 0.005, respectively) (Tables 2 and 3).

**Table 2:** Comparisons of optical coherence tomography and angiography parameters between chronic hypertension and normal control groups.

	Group A *n* = 45	Group C *n* = 50	*p*-value^*^
Vessel density 1 mm center (mean ± SD, mm^−1^)	8.0 ± 1.1	9.0 ± 1.7	0.003
Vessel density inner ring (mean ± SD, mm^−1^)	20.5 ± 2.0	21.7 ± 1.1	0.001
Vessel density 3 mm full (mean ± SD, mm^−1^)	19.4 ± 1.4	20.1 ± 1.0	0.005
Perfusion density 1 mm center (mean ± SD)	0.14 ± 0.02	0.16 ± 0.03	0.005
Perfusion density inner ring (mean ± SD)	0.37 ± 0.04	0.40 ± 0.02	0.001
Perfusion density 3 mm full (mean ± SD)	0.35 ± 0.02	0.37 ± 0.01	0.002
Foveal avascular zone area (mean ± SD, mm^2^)	0.35 ± 0.05	0.30 ± 0.07	<0.001
Central foveal thickness (mean ± SD, µm)	264.1 ± 15.0	272.0 ± 16.4	0.020
Mean GC-IPL thickness (mean ± SD, µm)	79.1 ± 11.3	85.0 ± 11.2	0.024
RNFL thickness (mean ± SD, µm)	93.1 ± 7.8	96.7 ± 7.2	0.025

-GC-IPL = ganglion cell inner plexiform layer; RNFL = retinal nerve fiber layer.

-Group A = chronic hypertension group, Group C = older healthy group.

-^*^Student’s *t* test.

**Table 3:** Comparisons of optical coherence tomography and angiography parameters between relieved hypertension and normal control groups

	Group B *n* = 40	Group D *n* = 50	*p*-value^*^
Vessel density 1 mm center (mean ± SD, mm^−1^)	9.5 ± 2.8	11.5 ± 1.7	0.005
Vessel density inner ring (mean ± SD, mm^−1^)	20.5 ± 2.0	22.4 ± 1.1	0.001
Vessel density 3 mm full (mean ± SD, mm^−1^)	19.8 ± 1.6	21.8 ± 0.8	<0.001
Perfusion density 1 mm center (mean ± SD)	0.17 ± 0.04	0.20 ± 0.03	0.010
Perfusion density inner ring (mean ± SD)	0.39 ± 0.03	0.41 ± 0.02	0.001
Perfusion density 3 mm full (mean ± SD)	0.37 ± 0.03	0.39 ± 0.01	0.015
Foveal avascular zone area (mean ± SD, mm^2^)	0.36 ± 0.03	0.29 ± 0.06	0.001
Central foveal thickness (mean ± SD, µm)	253.4 ± 15.9	277.3 ± 17.3	<0.001
Mean GC-IPL thickness (mean ± SD, µm)	72.7 ± 10.5	85.75 ± 6.3	<0.001
RNFL thickness (mean ± SD, µm)	91.3 ± 9.0	98.2 ± 7.3	0.005

-GC-IPL = ganglion cell inner plexiform layer; RNFL = retinal nerve fiber layer.

-Group B = relieved hypertension group, Group D = younger healthy group.

-^*^Student’s *t* test.

In repeatability analysis, the ICCs of all OCTA parameters were above 0.8 (Table 4).

**Table 4:** Interclass correlation coefficient (ICC) for optical coherence tomography angiography parameters.

	First measured value	Second measured value	ICC
Vessel density 1 mm center (mean ± SD, mm^−1^)	9.3 ± 2.3	9.5 ± 2.0	0.903
Vessel density inner ring (mean ± SD, mm^−1^)	21.3 ± 1.8	21.3 ± 1.8	0.955
Vessel density 3 mm full (mean ± SD, mm^−1^)	20.0 ± 1.6	20.1 ± 1.5	0.894
Perfusion density 1 mm center (mean ± SD)	0.15 ± 0.04	0.17 ± 0.04	0.886
Perfusion density inner ring (mean ± SD)	0.38 ± 0.04	0.37 ± 0.04	0.901
Perfusion density 3 mm full (mean ± SD)	0.35 ± 0.03	0.37 ± 0.02	0.855
Foveal avascular zone (mean ± SD, mm^2^)	0.32 ± 0.01	0.33 ± 0.01	0.821

### Correlation analyses between OCT and OCTA parameters

In group A and B, inner retinal thickness were significantly correlated with the VD 3 mm full (GC-IPL and VD 3 mm full; Group A: r = 0.635, *p* = 0.001; Group B: r = 0.512, *p* = 0.025, respectively) (RNFL and VD 3 mm full; Group A: r = 0.397, *p* = 0.012; Group B: r = 0.529 *p* = 0.020, respectively); there were no significant correlation in groups C and D (all *p* above 0.05). Between inner retinal thickness and other OCTA metrics (VD inner ring, PD inner ring, and PD 3 mm center) correlation analyses showed a same trend (Group A and B; *p* = 0.05, Group C and D; *p* above 0.05) (Table 5).

**Table 5:** Linear regression analyses between optical coherence tomography and optical coherence tomography angiography parameters.

		Group A			Group B			Group C			Group D		
		r	β	p-value	r	β	p-value	r	β	p-value	r	β	p-value
GC-IPL	VD inner ring	0.639	2.746	**<0.001**	0.521	4.649	**0.022**	0.113	0.667	0.473	0.243	−1.825	0.301
	VD 3 mm full	0.635	2.838	**<0.001**	0.512	5.104	**0.025**	0.162	1.056	0.298	0.175	−1.311	0.460
	PD inner ring	0.569	143.148	**<0.001**	0.583	282.042	**0.009**	0.199	77.362	0.201	0.316	−155.945	0.174
	PD 3 mm full	0.568	148.203	**<0.001**	0.614	334.690	**0.005**	0.227	93.918	0.143	0.269	−134.218	0.251
RNFL	VD inner ring	0.398	1.733	**0.012**	0.614	7.095	**0.005**	0.109	0.842	0.488	0.216	−2.105	0.361
	VD 3 mm full	0.397	1.800	**0.012**	0.529	6.831	**0.020**	0.115	0.982	0.461	0.134	−1.302	0.574
	PD inner ring	0.346	88.187	**0.031**	0.764	478.644	**<0.001**	0.235	119.360	0.130	0.306	−196.150	0.190
	PD 3 mm full	0.347	91.713	**0.031**	0.716	505.762	**0.001**	0.253	136.525	0.102	0.230	−149.477	0.328

-GCIPL = ganglion cell-inner plexus layer; RNFL = retinal nerve fiber layer; VD = vessel density; PD = perfusion density.

- Group A = chronic hypertension group, Group B = relieved hypertension group, Group C = older healthy group, Group D = younger healthy group.

- Boldface numbers indicate statistically significant differences at p < 0.05.

## Discussion

Hypertension is the fourth greatest risk factor for all deaths worldwide and a major cardiovascular risk factor^1,2^. Increased systemic arterial pressure and peripheral vascular resistance trigger functional and structural, macrovascular and microvascular alterations in the brain, heart, kidneys, and eyes. Rizzoni *et al*.^14^ reported that the monitoring of retinal circulation provides an easily accessible and noninvasive method to study structural alterations in larger arterioles using scanning laser Doppler flowmetry. Recent advances in SD-OCT and OCTA have allowed not only more detailed observation of retinal structure but also retinal microcirculation function.

In the present study, which was consistent with our previous study, the chronic HTN and relieved HTNR groups showed a significant thinning of CMT, GC-IPL, and RNFL compared to the normal control group^9,10^. In the previous study, we hypothesized that microvascular abnormalities may affect retinal thickness in patients with chronic hypertension and severe hypertension. Recently, the development of OCTA provided a clue of this hypothesis. In this study, the chronic HTN and relieved HTNR groups showed significantly decreased VD and PD, and significantly increased FAZ area compared to the normal control group. In addition, the chronic HTN and relieved HTNR groups showed significant correlation between OCTA and OCT parameters, unlike normal control group. Therefore, it can be deduced that chronic high blood pressure or previous episodes of high blood pressure affects retinal microcirculatory structure and function, which finally affects retinal thickness.

However, it can be supposed that inner retinal thinning may damage to microcirculation in vice versa. Malignant hypertensive patients undergo hypertensive choroidopathy which is related to choroidal ischemia^15,16^. Cotton-wool spots, which is frequently shown in grade IV HTNR, have been hypothesized to develop secondary to obstruction of a retinal arteriole with resultant ischemia and triggered permanent reductions in RNFL^17–19^. Additionally, in grade IV HTNR, the RNFL thickness and CMT at the time of initial diagnosis were increased due to optic disc edema and retinal swelling; and decrease of retinal thicknesses occurred over time^9^. Therefore, reduction of retina thickness in relieved HTNR group may apparently be the results of changes in microcirculation due to acute severe hypoxic damage.

The relationship between microcirculation and retinal thickness is also unclear in chronic hypertension group. Tables 2 and 3 show a different *p*-value trend between the chronic hypertension group and relived HTNR group. In chronic hypertension group, the *p* value of OCTA metrics is smaller than that of OCT metrics (OCTA metrics; *p* < 0.005, OCT metrics; *p* = *0*.*020~*0.025); in relieved HTNR group, all the *p* values of metrics are below 0.005. Table 5 also shows a different r value trend between the chronic hypertension group and relieved HTNR group in correlation of RNFL and OCTA metrics (Group A; r = 0.347~0.398, Group B; r = 0.529~0.716). This suggests that acute hypoxic ischemia due to high blood pressure has a more pronounced effect on microcirculation and retinal thickness than chronic hypertension, and there is a stronger correlation between the microcirculation and retinal thickness than chronic hypertension. Although OCTA shows reduced retinal perfusion in the hypertensive patients without HTNR changes in fundus examination, we can assume that there might be another unknown factor between the decrease in microcirculation and retinal thickness in chronic hypertension group. So, we need a prospective time serial study to know the more specific pathophysiology.

The retina may reflect the microvascular network in different vascular beds, acting as a surrogate marker of systemic microvascular function and resistance. In addition, microcirculatory damage in the retina appears to represent a systemic macrovascular condition^20^, as indicated by the relationship between macrovascular alterations and vascular remodeling in the retinal circulation, which is closely related to the cerebral microcirculation^21^. There have been many reports that retinal microvascular changes caused by hypertension play an important role in predicting risk and mortality for cardiovascular diseases^6,22–24^. However, there is no standardized assessment tool for retinal examination. OCTA may be utilized for assessment of cardiovascular risk in hypertension patients. This novel diagnostic technique is fast, quantitative, non-invasive, and easy to perform. OCTA parameters, VD and PD, may act as a surrogate marker of retina microcirculation. However, the systemic effects of these observations in patients with hypertension were not studied in detail. Further prospective studies are needed to determine whether retina vascular rarefaction has clinically relevant predictive value in OCTA.

While OCTA can produce angiograms that provide a noninvasive means of visualizing the retinal and choroidal vasculature, OCTA images suffer from certain imaging artifacts, such as pupil vignetting, defocusing, motion, shadow, projection, and segmentation error, which can affect the results^25,26^. Motion artifacts could be minimized by improving the eye tracking capability of OCTA systems. The FastTrack technique is available on Cirrus SD-OCTA, which uses a laser scanning ophthalmoscope to trace eye motion and guide OCT scanning, thus enabling motion-free OMAG angiograms^27^. However, motion artifacts were particularly troublesome in our study, even though we used eye tracking on OCTA systems. To minimize motion artifacts, we adopted the smallest scan to reduce inspection time, i.e., macular 3 × 3 mm, and used anesthetic eye drops and instructed the participants not to blink before each examination. Nevertheless, 16 eyes were excluded due to motion artifacts and autosegmentation errors, even though the signal strength exceeded 8. To confirm the reliability of the results, we performed the examinations twice and the ICCs were calculated. All values of OCTA parameters were above 0.8, which showed good repeatability of measurements.

This study had several limitations. First, we did not classify patients with hypertension according to the antihypertensive agents used in their treatment. Several studies have demonstrated regression of HTNR signs in response to blood pressure reduction, and indicated that regression patterns differ in response to different antihypertensive agents (e.g., angiotensin-converting enzyme inhibitors appear to have a more favorable effect on the retinal vasculature)^28,29^. Therefore, OCTA parameters may be affected by antihypertensive agents. However, it is difficult to determine the detailed medical history in HTN patients treated with antihypertensive agents for more than 10 years. Second, we did not analyze other factors (e.g., hyperlipidemia and smoking) that have been shown to induce ischemic damage in the retina. Third, some patients in the chronic hypertensive group could have experienced earlier hypertensive events causing retinal damage. In addition, the temporal precedence relationships between microcirculation of retina and retinal thickness is unclear. So, further prospective long-term studies with larger sample sizes are needed. Finally, this study included only normal axial length, so it is difficult to apply this method to shorter (axial length < 23.6 mm) or longer eyes (axial length > 25.5 mm).

Despite the limitations outlined above, this study also had a number of strengths. This was the first cross-sectional study to analyze OCTA parameters in hypertension patients. We also evaluated the long-term progression of retinal ischemia by comparing data from chronic, well-controlled hypertension patients with those of normal controls, which allowed detection of retinal changes associated with chronic ischemia even in patients with well-controlled hypertension. We used only vascular images of the SCP in this study; this method is free from shadow and projection artifacts, which cause major problems in deep capillary plexus studies. Therefore, VD and PD of the SCP value in this study seemed to be more objective than other parameters, which would be clinically useful for analysis of hypertension retinopathy. However, further studies of the deep capillary plexus in hypertensive patients without projection and shadow artifacts are needed.

In conclusion, OCTA is fast, quantitative, non-invasive, and easy to perform for examining HTNR, and our findings indicated that VD and PD were decreased in chronic HTN and relieved HTNR patients compared to normal controls. VD and PD may reflect the retina microcirculation. Chronic high blood pressure or previous episodes of high blood pressure affect OCTA parameters. Considering the effect of HTN on the retinal microvasculature, care is required in the interpretation of OCTA parameters in various ophthalmic conditions, such as retinal disease, glaucoma and neuro-ophthalmic disease.

## Data Availability

Data supporting the findings of the current study are available from the corresponding author on reasonable request.
